# Genome Sequences of Two New Pandoravirus Strains Isolated from Brazil and France

**DOI:** 10.1128/mra.00131-22

**Published:** 2022-06-22

**Authors:** Djamal Brahim Belhaouari, Sihem Hannat, Rodrigo Rodrigues, Sarah Aherfi, Bernard La Scola, Julien Andreani

**Affiliations:** a Aix-Marseille Université, Institut de Recherche pour le Développement (IRD), Microbes, Evolution, Phylogeny and Infection (MEPHI), Marseille, France; b Institut Hospitalo-Universitaire (IHU)—Méditerranée Infection, Marseille, France; c Laboratório de Vírus, Departamento de Microbiologia, Instituto de Ciências Biológicas Universidade Federal de Minas Gerais, Belo Horizonte, Minas Gerais, Brazil; DOE Joint Genome Institute

## Abstract

Pandoraviruses are giant viruses of amoebas with a wide range of genome sizes (1.5 to 2.5 Mbp) and 1-μm ovoid viral particles. Here, we report the isolation, genome sequencing, and annotation of two new strains from the proposed family Pandoraviridae: Pandoravirus belohorizontensis and Pandoravirus aubagnensis.

## ANNOUNCEMENT

Pandoraviruses are giant viruses as large as bacteria and have more complex genomes than some eukaryotic organisms (1). Several pandoraviruses have been described using coculture on Acanthamoeba castellanii (2–7). Here, we report the complete genome sequences of two novel strains: Pandoravirus belohorizontensis, isolated from soil samples collected from the city of Belo Horizonte (–19.923249777699425, −43.93308441843789), and Pandoravirus aubagnensis, from water collected in the south of France (Mounoï Cavern, also called “Manon des Sources”; 43.274642, 5.777029). The two viruses were isolated following a procedure previously described by Khalil et al. (8). Briefly, samples were cocultured on Acanthamoeba castellanii. They were characterized using flow cytometry and electron microscopy and then were produced and purified for genome sequencing. Viral DNA was extracted using an EZ1 Advanced XL automated system (Qiagen, France). DNA paired-end libraries (2 × 250-bp) were constructed with 1 ng of each genome as input using the Nextera XT DNA kit (Illumina, Inc., San Diego, USA) and sequenced on the Illumina MiSeq instrument. The reads were then trimmed and filtered using Trimmomatic (9). The P. belohorizontensis genome was assembled using CLC Genomics Workbench v7.52. The genome was finished using MUMmer v3.0 with default parameters (10), followed by a genome scaffolder using a graph-based approach (11). The genome of P. aubagnensis was assembled using SPAdes (12) and joined into a single scaffold using scaffold_builder (13). The genome termini were verified using Mauve software (14) and by a BLASTn search of both genomes against the nonredundant nucleotide (nr/nt) database (15). The analysis of both genomes followed the same procedure with default parameters. tRNAs were predicted using tRNAscan-SE (16) and ARAGORN (17) software. Gene predictions were performed using GeneMarkS (18). Predicted proteins over 99 amino acids long were considered for further analysis. The predicted proteins were investigated for putative functions and domains using BLASTp searches (E values, <1E-03) against the nonredundant protein database and the Pfam protein families database (19) and using Delta-BLAST (20). Phylogenetic analysis was based on the DNA polymerase subunit B gene. Amino acid sequences were aligned using Muscle (21). The maximum likelihood method was used for tree construction on MEGA7 (22) with the Jones-Taylor-Thornton model for amino acid substitution. The collection and analysis of genetic data were partially or fully registered under SisGen permit number AC31840 and SISBIO license numbers 33326, 34293, and 80252 (Brazil).

The P. belohorizontensis genome was assembled into a single scaffold of 1,701,725 bp (average coverage, 223×) with 19 gaps of unknown length and a G+C content of 63.67%. The P. belohorizontensis genome was predicted to encode 1,059 proteins (mean size ± SD, 363 ± 248 amino acids). Of these, 883 (83.4%) have a homolog in the nr/nt database, and 176 (16.6%) are ORFans (open reading frames [ORFs] with no significant homolog in the nr/nt database).

The assembly of the P. aubagnensis genome provided a single scaffold of 1,816,783 bp (average coverage, 198×) with 6 gaps of estimated length and a G+C content of 58.02%. A total of 1,217 proteins were predicted (mean size ± SD, 345 ± 244 amino acids). Of these, 907 (74.6%) have a homolog in the nr/nt database, and 309 (25.4%) are ORFans. tRNA prediction showed that both genomes encode a single proline tRNA.

**FIG 1:**
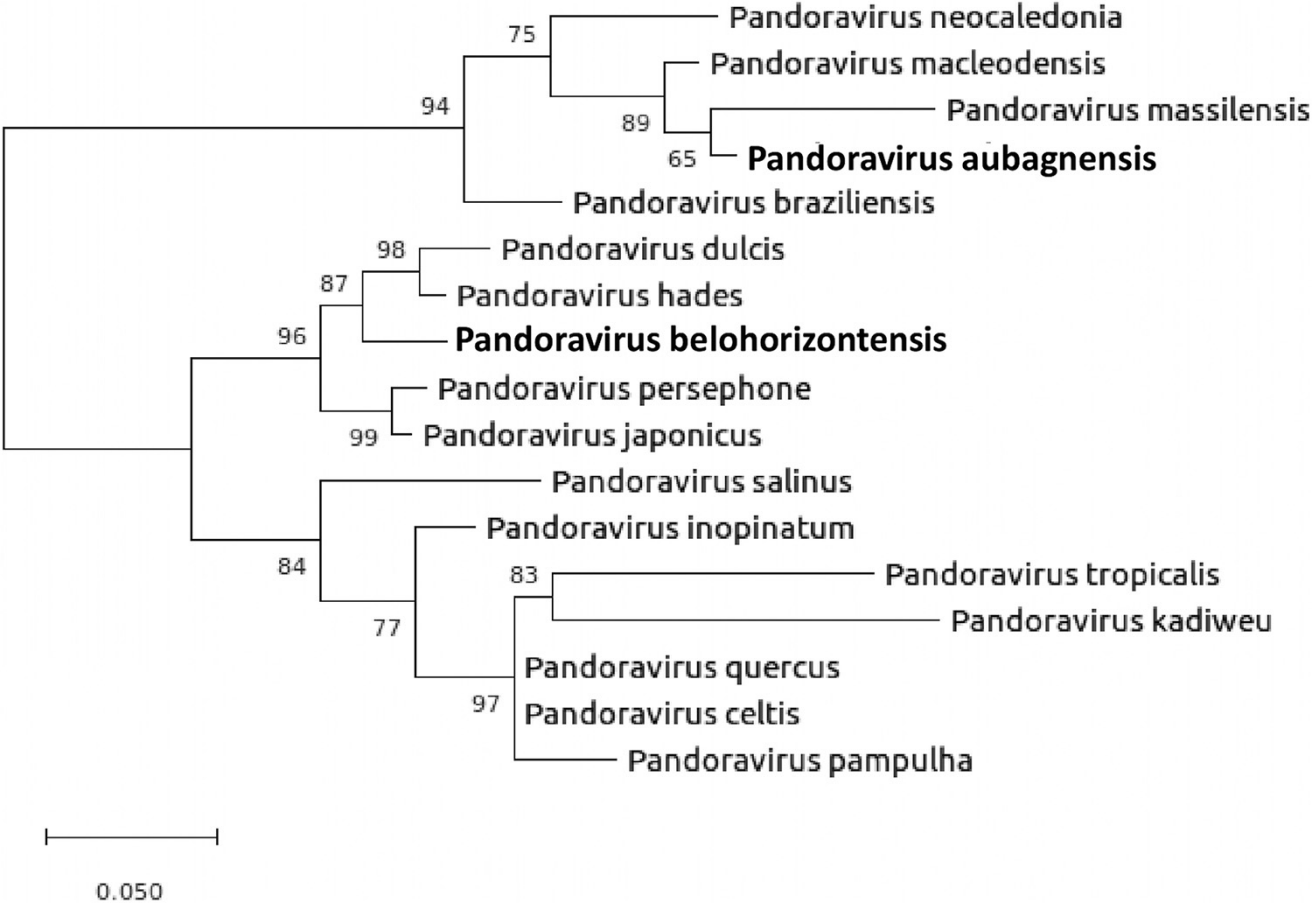
Phylogenetic reconstruction based on amino acid sequences of the DNA polymerase B subunit of Pandoravirus. The phylogenetic tree was built using the maximum likelihood model with 1,000 bootstrap replicates. The Pandoravirus kadiweu, P. tropicalis, P. pampulha, P. hades, and P. persephone sequences are partial predicted proteins (scale bar indicates 0.05 substitutions/site).

Phylogenetic analysis revealed that the two isolates were different from each other and clustered with previously described Pandoravirus lineages (Fig. 1).

### Data availability.

The genome sequences of Pandoravirus belohorizontensis and Pandoravirus aubagnensis have been deposited at NCBI GenBank under the accession numbers MZ420562 and MZ420563 and the annotation and SRA data under the SRA accession numbers SRR17644538 and SRR17635305, respectively. 
